# TFE3 fusions escape from controlling of mTOR signaling pathway and accumulate in the nucleus promoting genes expression in Xp11.2 translocation renal cell carcinomas

**DOI:** 10.1186/s13046-019-1101-7

**Published:** 2019-03-08

**Authors:** Xiaoqin Yin, Bo Wang, Weidong Gan, Wenyuan Zhuang, Zou Xiang, Xiaodong Han, Dongmei Li

**Affiliations:** 1 Immunology and Reproduction Biology Laboratory & State Key Laboratory of Analytical Chemistry for Life Science, Medical School, Nanjing University, Nanjing, 210093 Jiangsu China; 2 Jiangsu Key Laboratory of Molecular Medicine, Nanjing University, Nanjing, 210093 Jiangsu China; 3 Department of Urology, Affiliated Drum Tower Hospital of Medical School of Nanjing University, Nanjing, 210008 Jiangsu China; 4 Department of Health Technology and Informatics, Faculty of Health and Social Sciences, The Hong Kong Polytechnic University, Hung Hom, Kowloon Hong Kong, China

**Keywords:** TFE3, mTOR, Subcellular localization, Nuclear retention, Lysosome, Transcription factor

## Abstract

**Background:**

Xp11.2 translocation renal cell carcinoma (tRCC) is mainly caused by translocation of the TFE3 gene located on chromosome Xp11.2 and is characterized by overexpression of the TFE3 fusion gene. Patients are diagnosed with tRCC usually before 45 years of age with poor prognosis. We investigated this disease using two tRCC cell lines, UOK109 and UOK120, in this study.

**Methods:**

The purpose of this study was to investigate the pathogenic mechanism of TFE3 fusions in tRCC based on its subcellular localization, nuclear translocation and transcriptional activity. The expression of TFE3 fusions and other related genes were analyzed by quantitative reverse transcription PCR (qRT-PCR) and Western blot. The subcellular localization of TFE3 was determined using immunofluorescence. The transcriptional activity of TFE3 fusions was measured using a luciferase reporter assay and ChIP analysis. In some experiments, TFE3 fusions were depleted by RNAi or gene knockdown. The TFE3 fusion segments were cloned into a plasmid expression system for expression in cells.

**Results:**

Our results demonstrated that TFE3 fusions were overexpressed in tRCC with a strong nuclear retention irrespective of treatment with an mTORC1 inhibitor or not. TFE3 fusions lost its co-localization with lysosomal proteins and decreased its interaction with the chaperone 14–3-3 proteins in UOK109 and UOK120 cells. However, the fusion segments of TFE3 could not translocate to the nucleus and inhibition of Gsk3β could increase the cytoplasmic retention of TFE3 fusions. Both the luciferase reporter assay and ChIP analysis demonstrated that TFE3 fusions could bind to the promoters of the target genes as a wild-type TFE3 protein. Knockdown of TFE3 results in decreased expression of those genes responsible for lysosomal biogenesis and other target genes. The ChIP-seq data further verified that, in addition to lysosomal genes, TFE3 fusions could regulate genes involved in cellular responses to hypoxic stress and transcription.

**Conclusions:**

Our results indicated that the overexpressed TFE3 fusions were capable of escaping from the control by the mTOR signaling pathway and were accumulated in the nucleus in UOK109 and UOK120 cells. The nuclear retention of TFE3 fusions promoted the expression of lysosomal genes and other target genes, facilitating cancer cell resistance against an extreme environment.

**Electronic supplementary material:**

The online version of this article (10.1186/s13046-019-1101-7) contains supplementary material, which is available to authorized users.

## Background

Xp11.2 translocation renal cell carcinoma (Xp11.2 tRCC), also known as TFE3-fusion associated RCC, was listed as a new entity in the 2004 WHO classification of renal tumors and was recently included in the MiT (microphthalmia transcription factor) family tRCC as a new subset of RCC in the 2016 WHO classification [1, 2]. TFE3-fusion associated RCC is defined by several different translocations involving chromosome Xp11.2, including *ASPL, PRCC, NonO, CLTC, SFPQ1, LUC7L3, KHSRP, PARP14, DVL2,* and *RBM10,* as well as unknown genes on chromosome 10 [3–8]. All these resulted in gene fusions involving the Transcription Factor Binding to IGHM Enhancer 3 (*TFE3*) gene and Xp11.2 tRCC is characterized by overexpressed TFE3 fusion gene and affecting patients younger than 45 years with poor prognosis.

MiT family of transcription factors which encodes four distinct genes, including *MITF*, *TFEB*, *TFE3*, and *TFEC,* contains the basic helix-loop-helix (bHLH) structure and is capable of recognizing the transcription initiation or E-box (Ephrussi boxes) sites (CANNTG) in the genome. More recently, MITF, TFEB, and TFE3 have been identified as regulators of lysosomal function and metabolism. They can recognize numerous lysosomal and autophagy genes with one or more 10-base pair motifs (GTCACGTGAC) termed as Coordinated Lysosomal Expression and Regulation (CLEAR) elements, which in turn promotes gene transcription [9, 10]. To respond to the changes in the levels of nutrients within cells, TFE3 can regulate its intracellular distribution through activation or inactivation in an mammalian target of rapamycin complex 1 (mTORC1)-dependent manner [10, 11]. In fully fed cells, TFE3 is recruited to the lysosomal surface, where TFE3 undergoes mTORC1-dependent phosphorylation through interaction with active Rag GTPases. Active mTORC1 phosphorylates TFE3 at serine 321 (Ser321) residue to create a binding site for the cytosolic chaperone 14–3-3. Interaction with 14–3-3 proteins results in sequestration of TFE3 in the cytosol [10, 12, 13]. Conversely, when nutrients are scarce, inactivation of mTORC1, together with Ser321 dephosphorylation, prevents TFE3 proteins from binding to 14–3-3, resulting in the rapid accumulation of TFE3 in the nucleus. TFE3 thus regulates in the nucleus the expression of targeted genes, such as 4EBP3 which can repress translation initiation. Furthermore, depletion of endogenous TFE3 entirely abolishes the response of cells to starvation and overexpression of TFE3 triggers lysosomal exocytosis [10].

In Xp11.2 tRCC, the encoded fusion TFE3 proteins retains the bHLH structure for DNA binding, suggesting that these overexpressed fusion TFE3 proteins may function as oncogenic transcription factors. Up to now, the most widely accepted model that accounts for TFE3-fusion-mediated oncogenesis argues for the availability of a constitutively active promoter leading to an abnormal TFE3 transcriptional activity [3, 14, 15]. A multiple of molecular pathways well-implicated in carcinogenesis are regulated in part by the TFE3 protein, including activation of TGF-β and the ETS transcription factors, E-cadherin expression, CD40L-dependent lymphocyte activation, folliculin signaling, and mTORC1 signaling [16–19]. However, the intracellular distribution and specific functions of TFE3-fusion proteins in Xp11.2 tRCC still remain unknown, and thus, there is no effective treatment for patients with this RCC subtype.

In this study, we focused on two Xp11.2 tRCC cell lines, UOK109 from *NonO-TFE3* fusion and UOK120 from *PRCC-TFE3* fusion tRCC, to explore intracellular distribution of the fusion TFE3 proteins and possible pathogenic mechanisms as a result of changes in intracellular localization of the fusion TFE3 proteins. We found that highly overexpressed TFE3 fusions showed strong nuclear accumulation in Xp11.2 tRCCs and were capable of escaping from the control by the mTOR signaling pathway. Furthermore, the nuclear retention of TFE3 fusions could regulate the expression of lysosomal biogenesis genes, thus promoting cancer cells resistance against an extreme environment.

## Methods

### Cell culture

The cell lines used in this study included 786–0 human kidney adenocarcinoma cells (ATCC® CRL-1932™), HK-2 human kidney cortex/proximal tubule (ATCC® CRL2190™), and the renal carcinoma cell lines UOK120 and UOK109 (gifts of Dr. W. Marston Linehan, National Cancer Institute, Bethesda, MD). The UOK120 and UOK109 cell lines were derived from primary papillary cell carcinoma as described [20], and were derived from tumors arising in a 30- and a 39-year-old male, respectively. Cells were cultured in DMEM (Gibco, Grand Island, NY) supplemented with 10% FBS (Gibco) and 1% penicillin/streptomycin (Invitrogen, Carlsbad, CA).

### Antibodies

The antibodies used in this study included: mouse anti-GAPDH (Santa Cruz Biotechnology, sc-365,062), goat anti-TFE3 (Santa Cruz Biotechnology, sc-5958), rabbit anti-TFE3 (Abcam, ab93808), mouse anti-4EBP3 (Santa Cruz Biotechnology, sc-134,232), rabbit anti-pS6 (Cell Signaling Technology, 2215), rabbit anti-RPS6 (Abcam, ab40820), rabbit anti-mTOR (Cell Signaling Technology, 2972), rabbit anti-14-3-3 β/α (Cell Signaling Technology, 9636), rabbit anti-14-3-3 γ (Cell Signaling Technology, 5522), rabbit anti-14-3-3 ζ/δ (Cell Signaling Technology, 7413), rabbit anti-14-3-3 ε (Cell Signaling Technology, 9635), rabbit anti-14-3-3 τ (Cell Signaling Technology, 9638), rabbit anti-14-3-3 η (Cell Signaling Technology, 5521), rabbit anti-LAMP2 (Cell Signaling Technology, 49,067), rabbit anti-β-Tubulin (Cell Signaling Technology, 2146), HRP-conjugated goat anti-rabbit (Cell Signaling Technology, 7074), goat anti-mouse secondary antibody (Boster, BA1050), rabbit anti-goat secondary antibody (Boster, BA1060), Alexa Fluor 488-conjugated goat anti-rabbit (Abcam, ab150077), and Alexa Fluor 594-conjugated donkey anti-goat secondary antibodies (Invitrogen, A-11058).

### Immunofluorescence

Cells were grown on coverslips and fixed in 4% formaldehyde (Electron Microscopy Sciences) diluted in PBS for 15 min at room temperature. Slides were washed 3 times with PBS and permeabilized for 20 min in blocking buffer containing 0.1% saponin (Sigma Aldrich, St Louis), 0.02% sodium azide, and 3% bovine serum albumin (BSA) in PBS. Primary and secondary antibodies were incubated in blocking buffer for 1 h at room temperature. Slides were washed in blocking buffer and PBS after primary and secondary antibodies. Coverslips were then mounted with the Prolong Gold antifade reagent with DAPI (Invitrogen). Fluorescent images were examined and photographed using confocal microscopy (Olympus FV10i, Tyoko, Japan).

### Chromatin immunoprecipitation

Cells were cultured and subsequently crosslinked in 1% formaldehyde (Thermo Scientific, Carlsbad, CA, 28906) and processed according to the protocol from the Pierce™ Agarose ChIP Kit (Thermo Scientific, 26,156). Essentially, cells were lysed with Buffer 1 and pelleted. Pellets were resuspended in Buffer 1 and homogenized on ice and pelleted. Next, pellet was resuspended in Buffer 2. The chromatin fraction was sheared by sonication in 1.5 ml siliconized micro-centrifuge tubes. A 10-μg aliquot of DNA was reverse crosslinked to assess chromatin size. 500 μg of the resulting sheared chromatin samples were cleared overnight at 4 °C. Also, TFE3 (10 μg/sample) and nonspecific goat IgG antibody (for background control) were incubated with magnetic beads overnight and washed with Buffer 3. Pre-cleared chromatin samples were added to washed beads and incubated at 4 °C overnight. Beads were washed with Buffer 3 and then eluted and reversed crosslink with Buffer 4 at 65 °C overnight. The promoter region of human 4EBP3 was amplified from immunoprecipitated genomic DNA with primers for amplification of − 522 to − 352 region: forward CTTAGCCTCCCAAAGTGCTG and reverse GCCAAAGTCACACATCTTGC; primers for amplification of − 129 to + 27 region: forward GGCTGGCTTCCTAGCAGATA and reverse GGCGTTGAGGTCGAGGAG; and primers for amplification of + 449 to + 639 region: forward GGCTGGCTTCCTAGCAGATA and reverse GGCGTTGAGGTCGAGGAG.

### ChIP-seq analyses

ChIP was performed in UOK109 (3 × 10^8^) and UOK120 (3 × 10^8^) cells using the TFE3 antibody. ChIP-seq data were obtained using an Illumina Hiseq 2000 sequencer (Illumina, San Diego, CA) and sequencing reads were de-multiplexed by Illumina pipeline [21]. ChIP-seq data were mapped to the human genome (hg19) using Bowtie algorithm with up to 2 mismatch allowed and reads mapping to more than 20 locations along the genome were discard. ChIP-seq data generated form IgG were used against the sample data in calling enriched regions and to control for false discovery rate (FDR). Peaks were called using MACS version 2, with q-value to 0.05 [22]. Gene ontology (GO) terms were categorized based on cell localization and the biological process. The *P* values were obtained using DAVID Bioinformatics Resources 6.7. E-box sequence and distance from transcription start sites were analyzed using UCSC Genome Bioinformatics software.

### Luciferase reporter assay

The 4EBP3 promoter was obtained from the GenBank and was cloned into the pGL4.10 vector (Obio, Shanghai, China, H6119). Cells were transfected with a firefly luciferase-containing reporter plasmid (4EBP3 promoter) and Renilla luciferase-containing plasmid as an internal control. Relative activity of firefly luciferase to Renilla luciferase was determined using the Dual-Glo luciferase assay system. Cells were seeded in 24-well plates followed by transfection with 0.1 μg/well using Lipofectamine 2000 (Invitrogen).

### Western blots

Total protein was isolated from cells following various treatments. Cells were washed three times with PBS and lysed in ice cold extraction buffer (50 mM Tris-HCl pH 7.4, 150 mM NaCl, 1% NP, 0.1% SDS, and 1× protease inhibitor cocktail). Whole cell lysates were centrifuged at 12000 g at 4 °C. Soluble fractions were mixed with 5× loading buffer and heated at 100 °C for 5 min. Protein concentration was determined by the Bradford method. Proteins were separated using SDS-PAGE and the PVDF membrane (Roche, Basel, Switzerland) by standard procedures. Blots were blocked for 1 h at room temperature in TBS with 0.05% Tween 20 (Sigma Aldrich) and 5% nonfat milk. Primary antibodies were incubated overnight at 4 °C in Tris Buffered Saline Tween (TBST) with 3% BSA (Sigma Aldrich). HRP-conjugated secondary antibodies were incubated 1 h at room temperature. Blots were washed with TBST 6 times, 10 min each, after both primary and secondary antibody incubation. Protein bands were visualized with an enhanced chemiluminescence detection kit and recorded on a radiographic film (Alpha Innotech, San Jose, CA).

### Gene knockdown

Cells were plated in 6-well plates and transfected with short hairpin RNA (shRNA) for human TFE3 (Obio, Y3619 and Y3620) and a non-target shRNA control (Obio, Y007) at a final concentration of 1 μg/well using Lipofectamine 2000 Transfection Regent (Invitrogen) followed by incubation for 48 h. Next, cells were passaged and analyzed for knockdown efficiency 72 h post-transfection. For viral infection, cells were cultured in 12-well plates and transfected with the TFE3 knockdown virus (Genechem, Shanghai, China, 54,157, 54,158 and 54,159) or a negative control virus (Genechem, CON077) using polybrene. 72 h after transfection, cells were infected at a MOI (multiplicity of infection) of 5 per well in 6-well plates. Puromycin was used to establish a stable cell line.

### RNA isolation and relative quantitative PCR

Total RNA was isolated from cells using Trizol reagent (Invitrogen) according to the manufacturer’s instruction. RNA was reverse-transcribed using Hiscript Q RT Supermix for qPCR (Vazyme, Nanjing, China, R122–01). RNA expression was quantified using the SYBR Green ER (Roche, Basel, Switzerland) according to the manufacturer’s instruction. Duplicate total RNA samples were prepared from control and various treated cells and analyzed in triplicate by real-time PCR analysis using a 7300 system (Applied Biosystems, Grand Island, NY). The Ct values were analyzed using the 2^-ΔΔCt^ method. Amplification of the reference endogenous gene GAPDH was used to normalize the sequence of interest. The primers for mRNAs were shown in Additional file 1: Supplement S1.

### Statistical analysis

All calculations and statistical analyses were performed using SPSS for windows version 13.0 (SPSS Inc., Chicago, IL, USA). One-way analysis of variance (ANOVA) was used to analyze the differences between groups, followed by Dunnett’s t-test. *P* < 0.05 was regarded as statistically significant.

## Results

### TFE3 fusions accumulate in the nucleus in Xp11.2 tRCCs

**Fig. 1:**
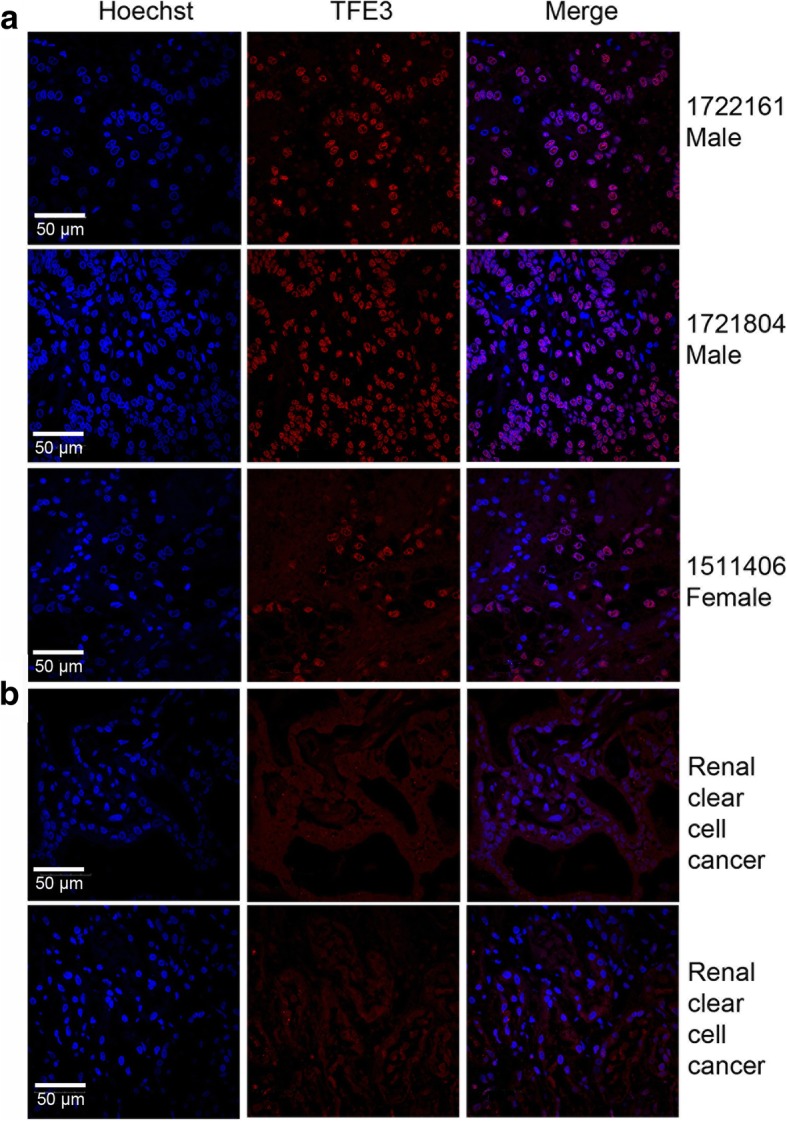
TFE3 fusion proteins are accumulated in the nucleus in Xp11.2 translocation renal cell carcinoma (tRCCs). **a**, **b** TFE3 proteins (red) were measured with immunofluorescence microscopy in the Xp11.2 tRCCs pathological sections and the renal clear cell carcinoma pathological sections. Nuclei were stained with Hoechst (blue). Scale bar, 50 μm

**Fig. 2:**
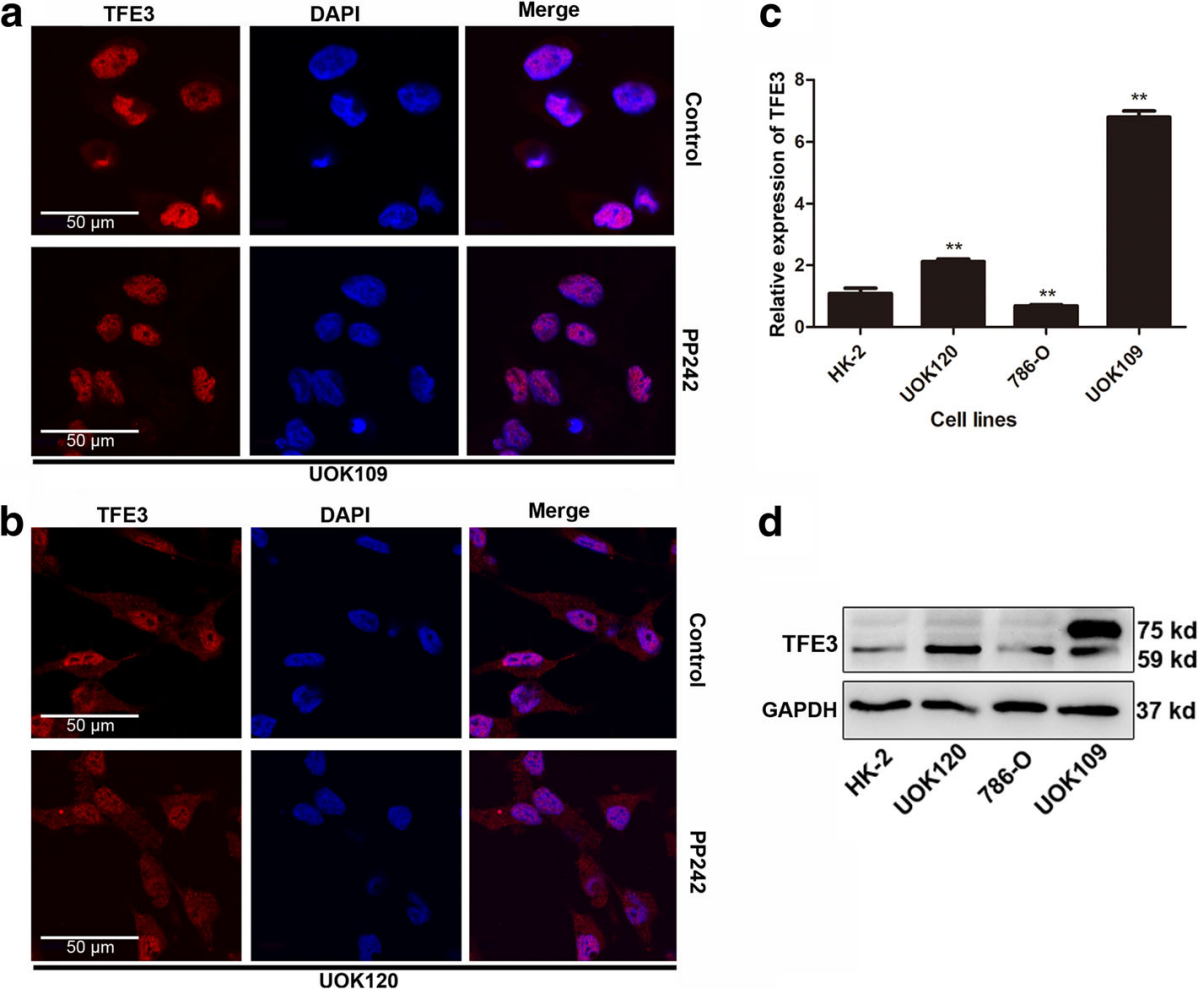
PP242 exerts no effect on the subcellular localization of TFE3 fusion proteins. Renal carcinoma cell lines including HK2, UOK109 and UOK120 were incubated in the absence (Control) or presence of PP242 (4 μM) for 24 h. **a**, **b** Cells were stained with TFE3 (red) followed by immunofluorescence photomicrographic analysis. **c**, **d** The expression of TFE3 was determined by q-PCR and Western blot. GAPDH was used as an internal control. *P < 0.05, **P < 0.01. Scale bar, 50 μm

Given that the intracellular distribution of TFE3 determines whether it can play a role in regulating cell metabolism as a transcription factor, we first aimed to investigate the intracellular distribution of TFE3 fusions. We found that TFE3 fusions accumulated in the nucleus in Xp11.2 RCC tumor samples (Fig. 1a), in contrast to few case of TFE3 localization within the nucleus in other renal clear cell cancer samples (Fig. 1b). These findings suggested that, once the TFE3 fused with other genes, it was mainly accumulated in the nucleus instead of the cytosol, which was further confirmed in UOK109 and UOK120 cells (Fig. 2a and b).

For UOK109 and UOK120 cells, we tested their fusion type, and found that UOK109 was *NonO-TFE3* and UOK120 was *PRCC-TFE3* (Additional file 2: Supplement S2), which was consistent with previous reports. Compared with none TFE3 fusion cells (786–0 and HK-2), the expression of fusion TFE3 genes increased significantly in UOK109 and UOK120 at both mRNA and protein levels (Fig. 2c and d).

According to the literature, active mTORC1 can phosphorylate TFE3 on Ser321, resulting in the creation of a binding site for the 14–3-3 proteins that TFE3 is sequestered in the cytosol without its transcriptional activity. To study the subcellular localization of the fusion TFE3 protein and its response to the mTORC1 signaling pathway, we introduced the mTOR inhibitor (PP242) into UOK109 and UOK120. After incubation with PP242 for 24 h, TFE3 fusions showed no nuclear translocation whereas nuclear retention was observed in the control cells left untreated (Fig. 2a and b). In contrast, in 786-O and HK-2 cells, wild-type TFE3 proteins showed a rapid translocation from cytosol to the nucleus following by treatment with PP242 (Additional file 2: Supplement S2).

### TFE3 fusions escape from the control of mTOR signaling pathway

#### TFE3 fusions fail to co-localize with LAMP2 in cytoplasm

**Fig. 3:**
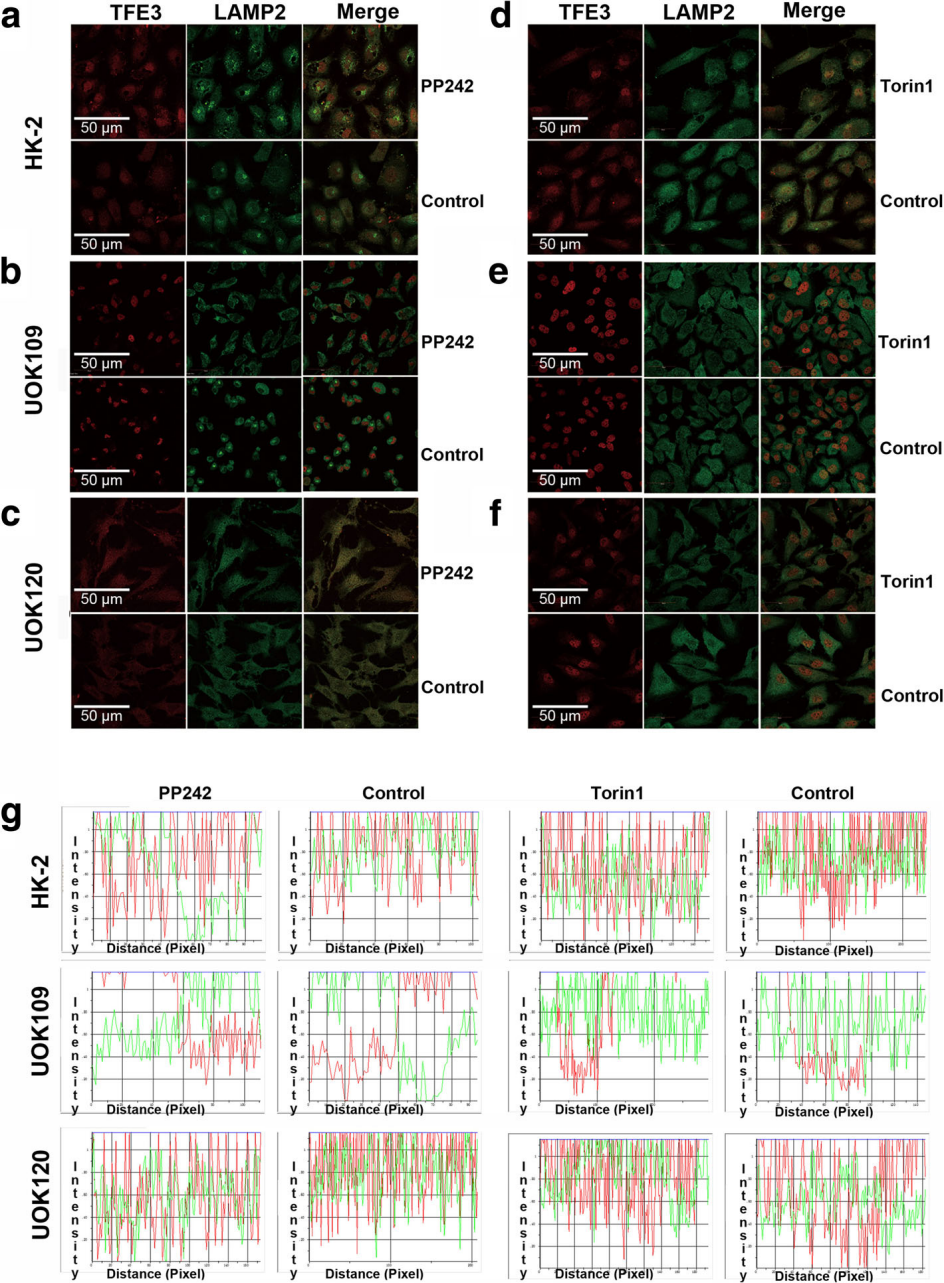
TFE3 fusion proteins fail to co-localize with the lysosomal protein LAMP2. Renal carcinoma cell lines including HK2, UOK109 and UOK120 were treated with PP242 (4 μM) or torin1 (4 nM) or left untreated (Control). **a** to **f** Cells were stained with antibodies against TFE3 (red) and LAMP (green) followed by immunofluorescence photomicrographic analysis. (**g**) The merging of the red and green fluorescence signals shown in a to f were quantified with Image-pro Plus. Scale bar, 50 μ

In the cytoplasm, phosphorylated TFE3 can be recruited to the lysosomal surface through direct interaction with active mTORC1 and this recruitment is critical for maintaining TFE3 sequestration in the cytosol. LAMP2 (lysosome-associated membrane protein 2) is a lysosomal surface marker. We found that wild-type TFE3 could co-localize with LAMP2, and incubation with a mTOR inhibitor (PP242 or Torin1) could separate LAMP2 from TFE3 in HK-2 (Fig. 3a and d). However, TFE3 fusions showed rare co-localization with LAMP2 in UOK109 and UOK120 cells, in which TFE3 fusions were mostly located in the nucleus and LAMP2 stayed at cytoplasm (Fig. 3b, c, e, f). And TFE3 fusions remained its nuclear retention after treatment with mTOR inhibitor. These data indicated that the subcellular localization of TFE3 fusions was capable of escaping from the control of mTOR signaling pathway and could not be recruited to the lysosomal surface.

#### Interaction between TFE3 fusions and the cytosolic chaperone 14–3-3 is decreased in UOK109 and UOK120 cells

**Fig. 4:**
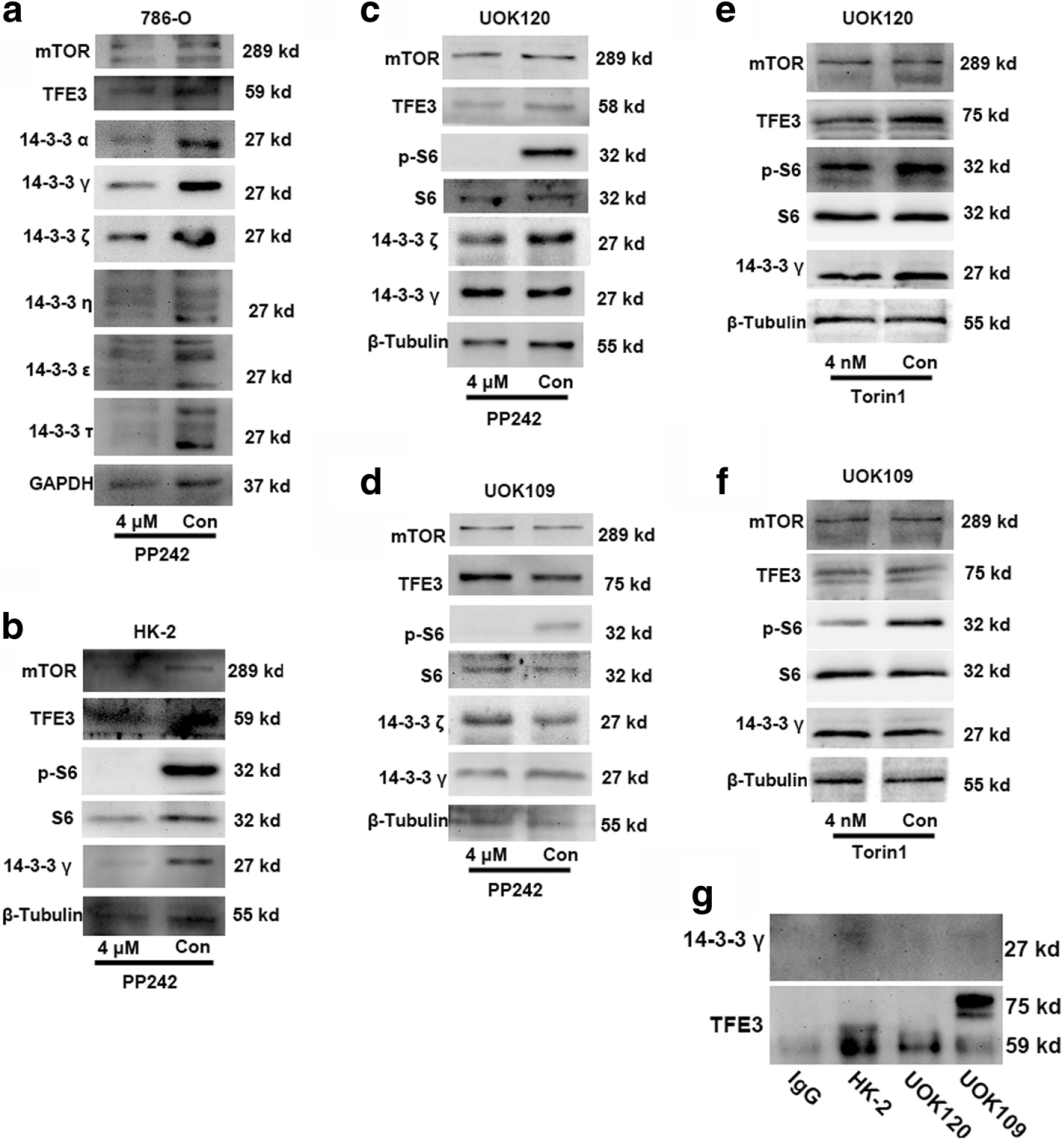
Binding between 14 and 3-3 proteins and TFE3 fusion proteins is reduced in the UOK109 and UOK120 cells. (**a**) 786-O cells were treated with PP242 (4 μM) or vehicle control (Con) for 24 h followed by immunoblot analysis of 14–3-3 proteins. GAPDH was used as an internal control. (**b**) HK-2 cells were treated with PP242 (4 μM) or vehicle control (Con) for 24 h followed by immunoblot analysis of 14–3-3 proteins. β-Tubulin was used as an internal reference. (**c**, **d**) UOK109 and UOK120 cells were treated with PP242 (4 μM) or vehicle control (Con) for 24 h followed by immunoblot analysis of 14–3-3 γ, 14–3-3 ζ, p-S6, S6 and mTOR proteins. (**e**, **f**) HK-2 cells were treated with Torin1 (4 nM) or vehicle control (Con) for 24 h followed by immunoblot analysis of 14–3-3 γ, phosphor-S6 (p-S6), S6 and mTOR proteins. (**g**) The binding between TFE3 and 14–3-3 γ was detected by co-immunoprecipitation (CO-IP). An irrelevant IgG was used as negative control

The 14–3-3 proteins have been identified as major binding partners of TFE3. The 14–3-3 proteins typically interact with their targets via short phosphoserine containing motifs. In order to determine whether 14–3-3 proteins were implicated in the control of TFE3 subcellular localization, we screened for candidates through the whole family of cytosolic chaperone 14–3-3 proteins. Three subtypes of 14–3-3 proteins, including 14–3-3α, 14–3-3γ, and 14–3-3ζ, were found to participate in TFE3 cytosol retention (Fig. 4a). Although the other three isoforms of the 14–3-3 proteins were also changed, multiple protein bands were revealed which implied that they could interact with other isoforms to form heterodimers. As shown in Fig. 4a and b, the 14–3-3γ was decreased apparently in 786–0 and HK-2 cells after treatment with the mTOR inhibitor. While there were no differences in UOK109 and UOK120 irrespective of treatment with the mTOR inhibitor (Fig. 4c-f). The Co-IP data suggested that the binding between TFE3 fusions and 14–3-3γ was reduced in UOK109 and UOK120 cells compared with in HK-2 cells (Fig. 4g). Together, these data indicated that TFE3 fusions failed to bind with the cytosolic chaperone 14–3-3γ which was able to induce the nuclear accumulation of TFE3 fusions.

#### Fusion segment of TFE3 proteins cannot translocate to nucleus

**Fig. 5:**
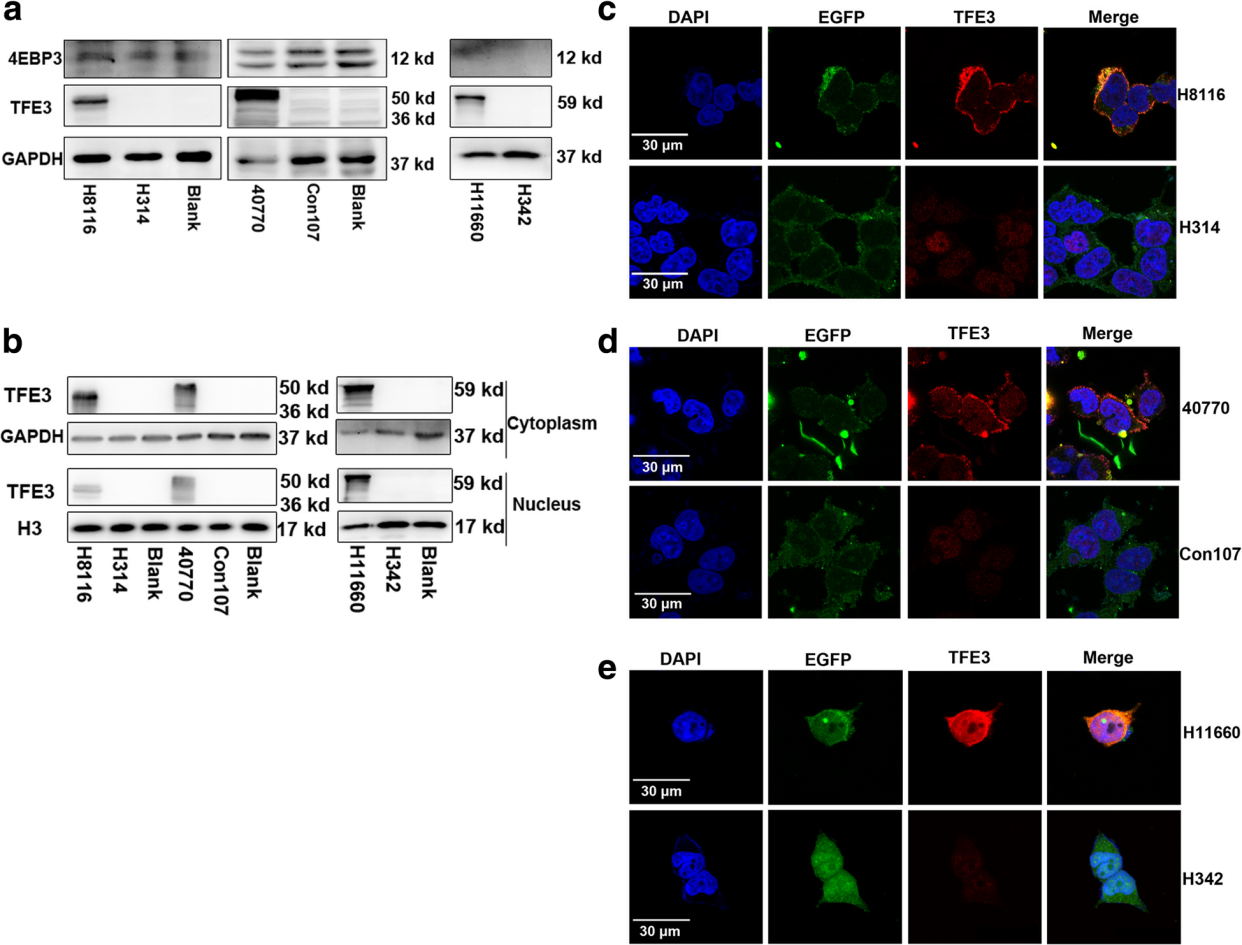
The fusion segments of TFE3 cannot be translocated to nucleus. (**a**) Expression levels of TFE3 and 4EBP3 were examined by immunoblot analysis. 293 T cells were transfected with plasmids H8116 (TFE3 296–575 aa), 40,770 (179–575 aa) or H11660 (full TFE3), each being compared to their relative controls by the transfection of negative control plasmids H314, Con107 and H342. (**b**) Expression levels of TFE3 in the cytoplasm and nucleus were examined by immunoblot analysis. Proteins expressed in the cytoplasm and nucleus were separated 48 h after transfection. GAPDH was used as a cytoplasmic internal control and H3 was shown as a nuclear internal control. (**c**) Cells were transfected with plasmids H8116 or H314. Expression of TFE3 (red) and EGFP (green) in 293 T cells was examined by immunofluorescence microscopy 48 h after transfection. Expression intensity of the plasmids in 293 T cells was examined based on EGFP fluorescence. Nuclei were stained with DAPI (blue). (**d**) Cells were transfected with plasmids 40,770 or Con107. Expression of TFE3 (red) and EGFP (green) in 293 T cells was examined by immunofluorescence microscopy 48 h after transfection. (**e**) Cells were transfected with plasmids H11660 or H342 for 48 h 48 h after transfection. Expression of TFE3 (red) and EGFP (green) in 293 T cells was examined by immunofluorescence microscopy. Scale bar, 30 μm

According to previous studies, the first 30 N-terminal residues of TFE3 are both necessary and sufficient for interaction with active Rag heterodimers which could bind the mTORC1 and recruit TFE3 to lysosomes [10]. The TFE3 fusion fragment in UOK109 were 296–575 amino acid longs at C-terminal and the fusion fragment in UOK120 was composed of the 179–575 amino acids at C-terminal. Based on these findings, we designed three types of expression plasmids containing TFE3 fused to EGFP including H8116 (TFE3 296-575aa), 40,770 (TFE3 179-575aa) or H11660 (full TFE3). The full TFE3 was used as a positive control. Western blot revealed that the TFE3 was dramatically increased in H11660 infected group and its target protein 4EBP3 was increased at the same time (Fig. 5a). However, in H8116 and 40,770 infected group, even TFE3 (296–575 aa) and TFE3 (179–575 aa) showed evidently increases. In contrast, the expression of 4EBP3 was not increased (Fig. 5a). TFE3 (296–575 aa) and TFE3 (176–575 aa) were mainly increased in the nucleus. Few of TFE3 protein segments were expressed in the cytoplasm as confirmed by Western blot (Fig. 5b). TFE3 (296–575 aa) and TFE3 (176–575 aa) were mainly localized in the cytoplasm together with EGFP in 293 T cells confirmed by immunofluorescence assays. Few TFE3 segments were localized in the nucleus (Fig. 5c and d). Whereas, the full TFE3 protein was localized both in the cytoplasm and the nucleus (Fig. 5e). All these data demonstrated that TFE3 fusion segments only expressed and were localized in the cytoplasm but not in the nucleus.

#### Inhibition of GSK3β increases the cytoplasm retention of TFE3 fusions

**Fig. 6:**
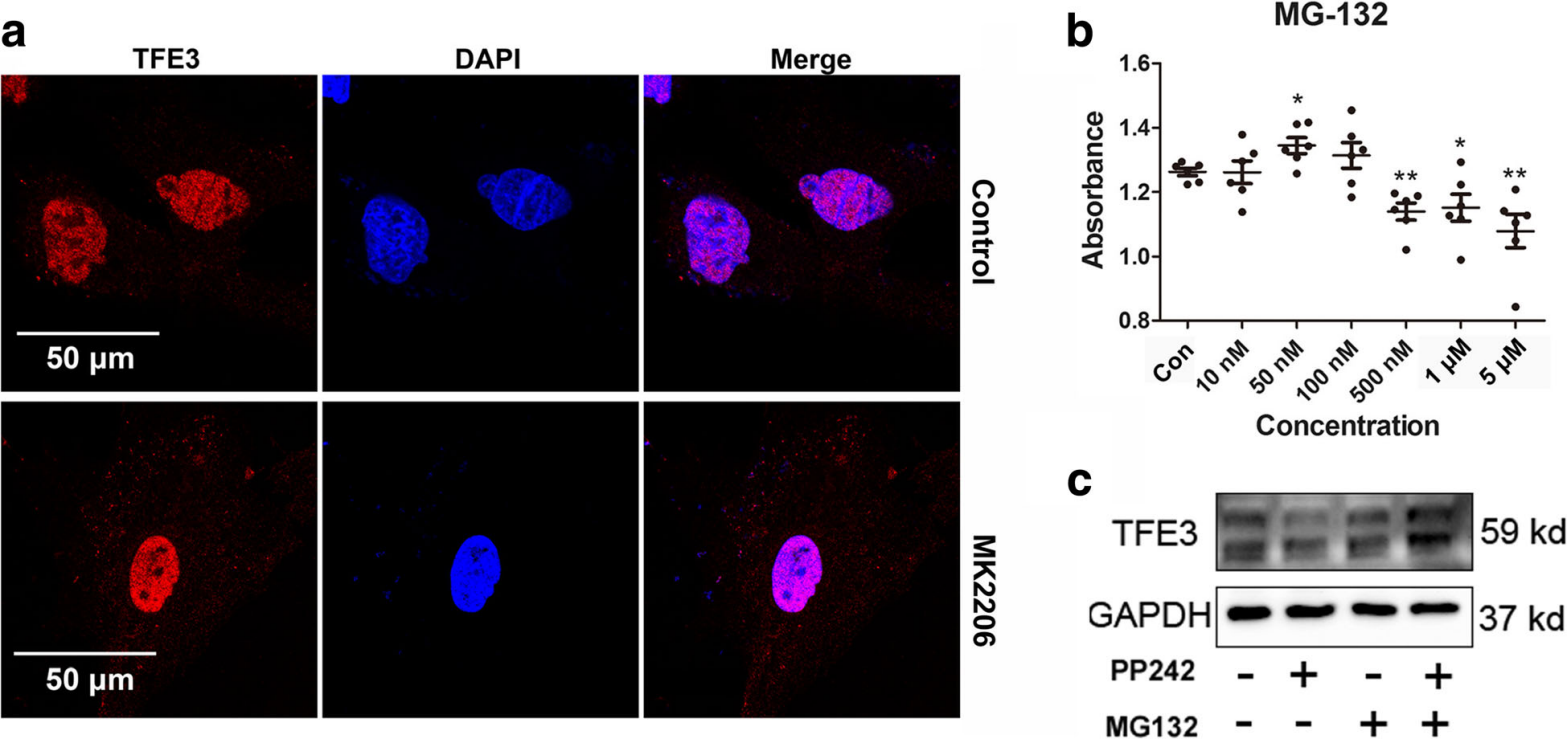
TFE3 fusion proteins are increased in the cytoplasm as a result of inhibition of the ubiquitination degradation or activation of Gsk3β. (**a**) UOK120 cells were treated with AKT inhibitor (MK2206, 5 μM) or vehicle control (Control) for 24 h. Expression levels of TFE3 (red) was examined by immunofluorescence microscopy. Nucleus were stained with DAPI (blue). Scale bar, 50 μm. (**b**) HK-2 cells viability were detected by CCK-8 after treatment with MG-132 or vehicle control (Con) for 24 h. (**c**) Expression levels of TFE3 were examined by immunoblot analysis. HK-2 cells were treated with PP242 (2 μM) or MG-132 (500 nM) for 24 h

Two residues (Ser134 and Ser138) in TFE3 are highly conserved in MiTF family, which could be phosphorylated by GSK3β leading to their cytoplasmic retention [23, 24]. In UOK109 and UOK120 cells, we found that only *PRCC-TFE3* fusions retained the two residues by sequence alignment (Additional file 3: Supplement S3). Our data showed that TFE3 fusions failed to be subject to the regulation by mTORC1 and accumulated in the nucleus in Xp11.2 tRCCs. In order to increase cytoplasmic retention of TFE3 fusions, we activated GSK3β through inhibiting AKT using MK2206 in UOK120 cells. It was revealed that TFE3 fusions were evidently increased in the cytoplasm (Fig. 6a). In addition, the uncoupled TFE3 in the cytoplasm could be dephosphorylated and degraded through ubiquitination. Next, we inhibited the ubiquitination degradation pathway by MG132 in HK-2 cells. The expression of TFE3 was increased and mTOR inhibition mediated reduction was recovered after treatment with MG132 (Fig. 6b and c). These data indicated that activating Gsk3β could lead to retention of TFE3 fusions in the cytoplasm which was accompanied with a reduction in its nuclear retention.

#### TFE3 fusions bind to the promoters of targeted-genes as native TFE3

To explore the molecular function of TFE3 fusions, we performed chromatin immunoprecipitation coupled to deep sequencing (ChIP-seq). We identified 4219 peaks in UOK109 cells and 1584 peaks in UOK120 cells (Additional file 4: Supplement S4). The predicted target genes in UOK109 and UOK120 cells were shown in Additional file 5: Supplement S5 and Additional file 6: Supplement S6. In order to gain an insight into the identification of the promoters occupied by TFE3 fusions, we used the web tool DAVID to perform GO analysis. Components associated with localization, organic substance transport, lytic vacuole organization, lysosome organization, and transport as shown in Table 1 significantly interacted with TFE3 fusions in UOK109 cells. The analysis of lysosomes demonstrated significant increases of the number of genes involved in the lysosome organization, endosome to lysosome transport and phagolysosome assembly, which suggested that TFE3 fusions could bind to the CLEAR element and regulate lysosomal biosynthesis. The analysis of KEGG pathway revealed that lysosomes were correlated with TFE3 (Table 2) which further confirmed *NonO-TFE3* fusions could still regulate lysosomal biogenesis genes as the wild-type TFE3. GO analysis in the UOK120 cells showed that components associated with the regulation of cellular macromolecule biosynthetic processes were significantly correlated with TFE3 fusions (Table 3). Different from UOK109 cells, the predicted gene functions mainly focused on the transcription processes and nutrient metabolic processes but not the lysosomal biogenesis in UOK120 cells. Furthermore, the MAPK signaling pathway and focal adhesion pathway showed significant correlation with *PRCC-TFE3* fusions by KEGG pathway analyses (Table 4).

**Table 1:** The GO analysis data of the target genes in UOK109

GOBPID	Term	Pvalue
GO:0051179	localization	2.02E-05
GO:0071702	organic substance transport	4.68E-05
GO:0007040	lysosome organization	8.18E-05
GO:0080171	lytic vacuole organization	8.18E-05
GO:0006810	transport	9.80E-05
GO:0051234	establishment of localization	0.000125094
GO:0071705	nitrogen compound transport	0.000309726
GO:0070482	response to oxygen levels	0.000391147
GO:0061025	membrane fusion	0.000394916
GO:0006687	glycosphingolipid metabolic process	0.000417151

**Table 2:** The KEGG pathway analysis of the target genes in UOK109

KEGGID	Term	Pvalue
4142	Lysosome	3.91E-06
604	Glycosphingolipid biosynthesis - ganglio series	0.002229366
520	Amino sugar and nucleotide sugar metabolism	0.002417655
760	Nicotinate and nicotinamide metabolism	0.013163582
5144	Malaria	0.013980843
524	Butirosin and neomycin biosynthesis	0.014139062
603	Glycosphingolipid biosynthesis - globo series	0.015695803
3440	Homologous recombination	0.022457622
5216	Thyroid cancer	0.025272857
511	Other glycan degradation	0.026898164

**Table 3:** The GO analysis data of the target genes in UOK120

GOBPID	Term	Pvalue
GO:2000112	regulation of cellular macromolecule biosynthetic process	6.88E-05
GO:0006355	regulation of transcription, DNA-templated	7.56E-05
GO:1903506	regulation of nucleic acid-templated transcription	8.70E-05
GO:2001141	regulation of RNA biosynthetic process	0.000100025
GO:0009889	regulation of biosynthetic process	0.000130783
GO:0010556	regulation of macromolecule biosynthetic process	0.000139177
GO:0051252	regulation of RNA metabolic process	0.000192021
GO:0006351	transcription, DNA-templated	0.000206295
GO:0031326	regulation of cellular biosynthetic process	0.000207896
GO:0097659	nucleic acid-templated transcription	0.000228788

**Table 4:** The KEGG pathway analysis of the target genes in UOK120

KEGGID	Term	Pvalue
4010	MAPK signaling pathway	0.034063246
4510	Focal adhesion	0.045985224

**Fig. 7:**
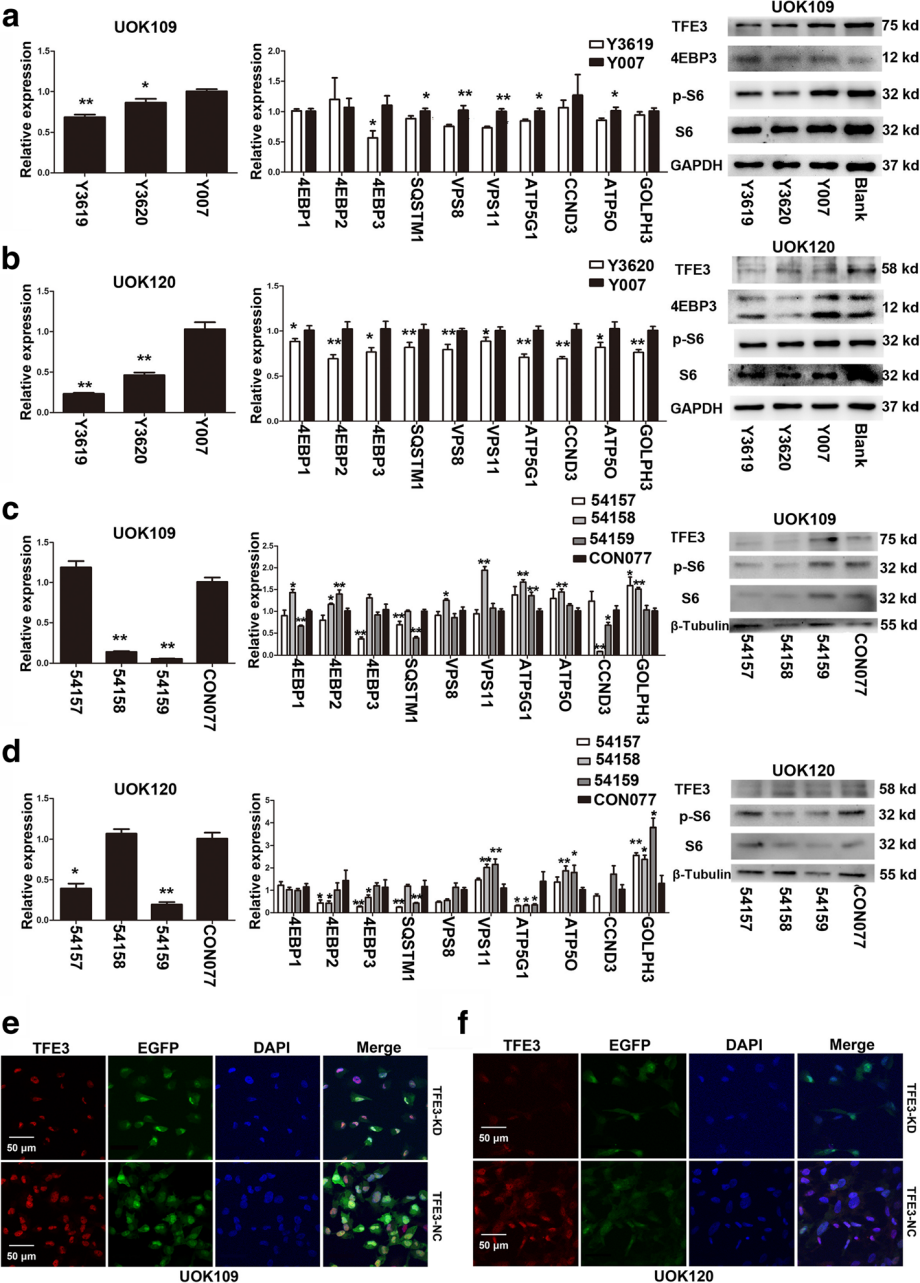
TFE3 fusions regulate lysosome biogenesis genes. The mRNA expression of TFE3, SQSTM1, VPS8, VPS11 ATP5G, ATP5O, 4EBPs and GOLPH3 were detected by q-PCR. Western blot was used to detect the expression of TFE3, 4EBP3, S6, and p-S6 on protein level. Immunofluorescence analysis were applied to confirm the subcellular localization of TFE3 fusion proteins. (**a**, **b**) UOK109 and UOK120 cells were transfected with TFE3 knockdown shRNA (Y3619 and Y3620) or negative control shRNA (Y007) for 48 h. mRNA levels of TFE3 and other related genes were examined by q-PCR. (**c**, **d**) UOK109 and UOK120 cells were transfected with TFE3 knockdown virus (54,157, 54,158 and 54,159) or negative control virus (CON077) for 72 h. mRNA levels of TFE3 and other related genes were detected with q-PCR. (**e**) UOK109 cells were transfected with 54,157 (TFE3-KD) or CON077 (TFE3-NC). After 72 h incubation, TFE3 fusions (red) were stained with antibodies and measured with immunofluorescence. Nucleus were stained with Hoechst (blue) and EGFP showed expression intensity of the virus. Scale bar, 50 μm. (**f**) UOK120 cells were transfected with 54,157 (TFE3-KD) or CON077 (TFE3-NC). After 72 h incubation, TFE3 fusions (red) were stained with antibodies and measured with immunofluorescence. Nucleus were stained with Hoechst (blue) and EGFP (green) showed expression intensity of the virus. Scale bar, 50 μm. Data were presented as the mean ± SEM. *P < 0.05, **P < 0.01. The unpaired two-tailed Student’s *t*-test was used to calculate *P* value

Since TFE3 fusions were significantly overexpressed and accumulated in the nucleus in UOK109 and UOK120 cells, we found that TFE3 fusions might also influence the expression of lysosomal genes or other target genes. In UOK109 and UOK120 cells, down-regulation of TFE3 fusions decreased the mRNA abundance of 8 out of 10 genes tested, including 4EBP3, SQSTM1, VPS8, VPS11, CCND3, ATP5G, ATP5O and GOLPH3. The expression of 4EBP3 at the protein level showed a significant decrease after TFE3 knockdown with plasmids or viral infection (Fig. 7a-d). In addition, the expression of S6 and p-S6 which are downstream of mTORC1 also showed an evident decrease. In TFE3 knockdown cells, the expression of TFE3 fusions was reduced, while the subcellular localization of TFE3 fusions remained unchanged in UOK109 and UOK120 cells (Fig. 7e and f). These data suggested that TFE3 fusions could bind to the CLEAR element as the wild-type TFE3 and could regulate the expression of lysosomal biogenesis genes.

**Fig. 8:**
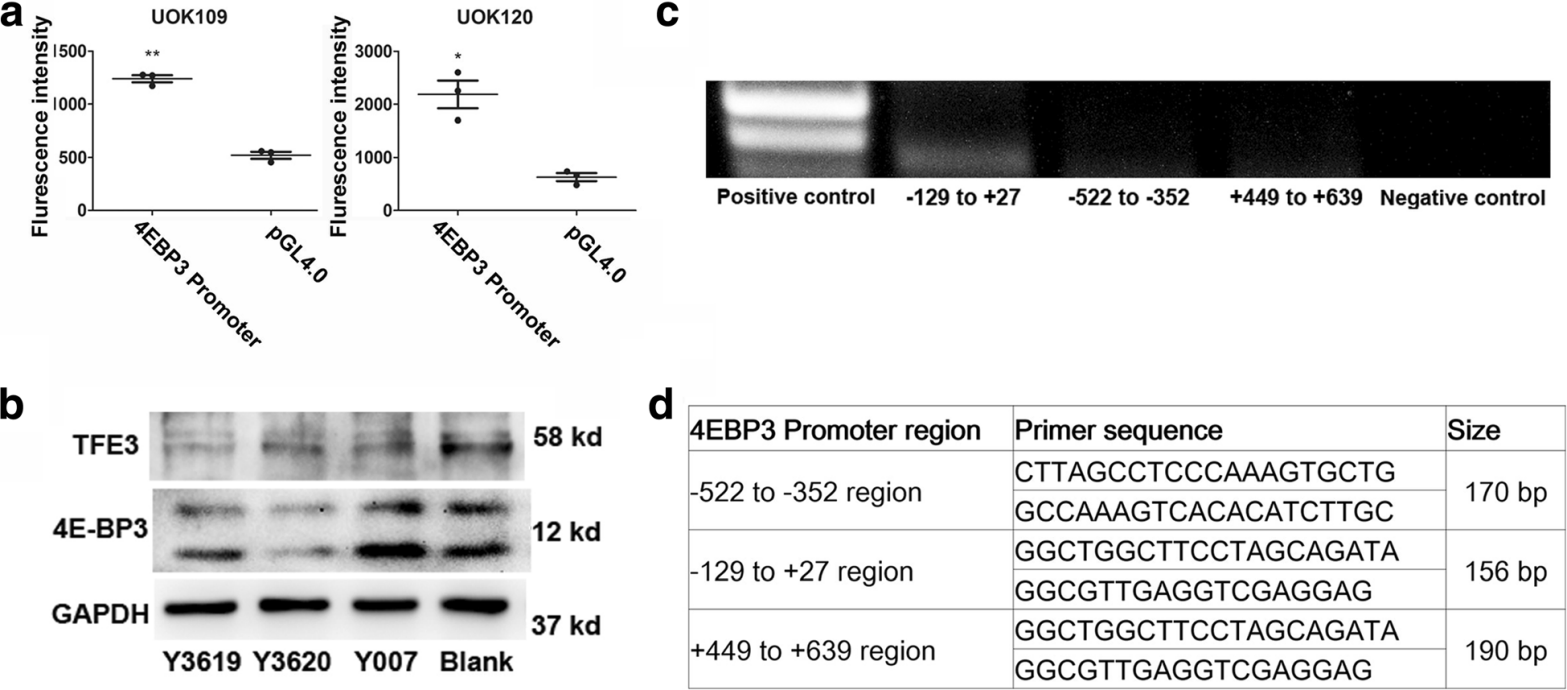
TFE3 fusions could bind to the promoter of 4EBP3. (**a**) The transcriptional activity of TFE3 fusions with different splicing variants was detected with luciferase reporter assay in UOK109 and UOK120 cells. Cells were transfected with 4EBP3 promoter expressing plasmids (4EBP3 Promoter) or negative control plasmids (pGL4.0) for 48 h. (**b**) Western blot was used to determine the expression of TFE3 and 4EBP3 after 48 h transfection of TFE3 knockdown shRNA (Y3169 and Y3620), negative control shRNA (Y007) or blank control (Blank). (**c**) The interaction between TFE3 fusions and 4EBP3 promoter was detected by Chromatin immunoprecipitation (ChIP). PCR was applied to detect the target DNA fragments. (**d**) The predicted target binding sites to 4EBP3 were showed in the table. Data were presented as the mean ± SEM. *P < 0.05, **P < 0.01. The unpaired two-tailed Student’s *t*-test was used to calculate *P* value

Furthermore, we chose 4EBP3 as a target gene to test the transcriptional activity of TFE3 fusions. To examine how the transcriptional induction of 4EBP3 was regulated by TFE3 fusions, we generated luciferase reporter plasmids containing the 4EBP3 promoter region. Luciferase assay data showed that TFE3 fusions could bind to the 4EBP3 promoter region and activate its transcription, which indicated that the TFE3 fusions remained its transcriptional activity (Fig. 8a and b). In addition, we performed a chromatin immunoprecipitation assay to determine whether TFE3 fusions directly binds to the 4EBP3 promoter at the E-box. The chromatin immunoprecipitation data showed that TFE3 fusions bind to the DNA segment containing the E-box (− 129 to + 27) of the 4EBP3 promoter, but not the sites from − 522 to − 352 or from + 449 to + 639 (Fig. 8c and d). Taken together, these results demonstrated that TFE3 fusions could mediate the transcriptional initiation of target genes just like the wild-type TFE3.

**Fig. 9:**
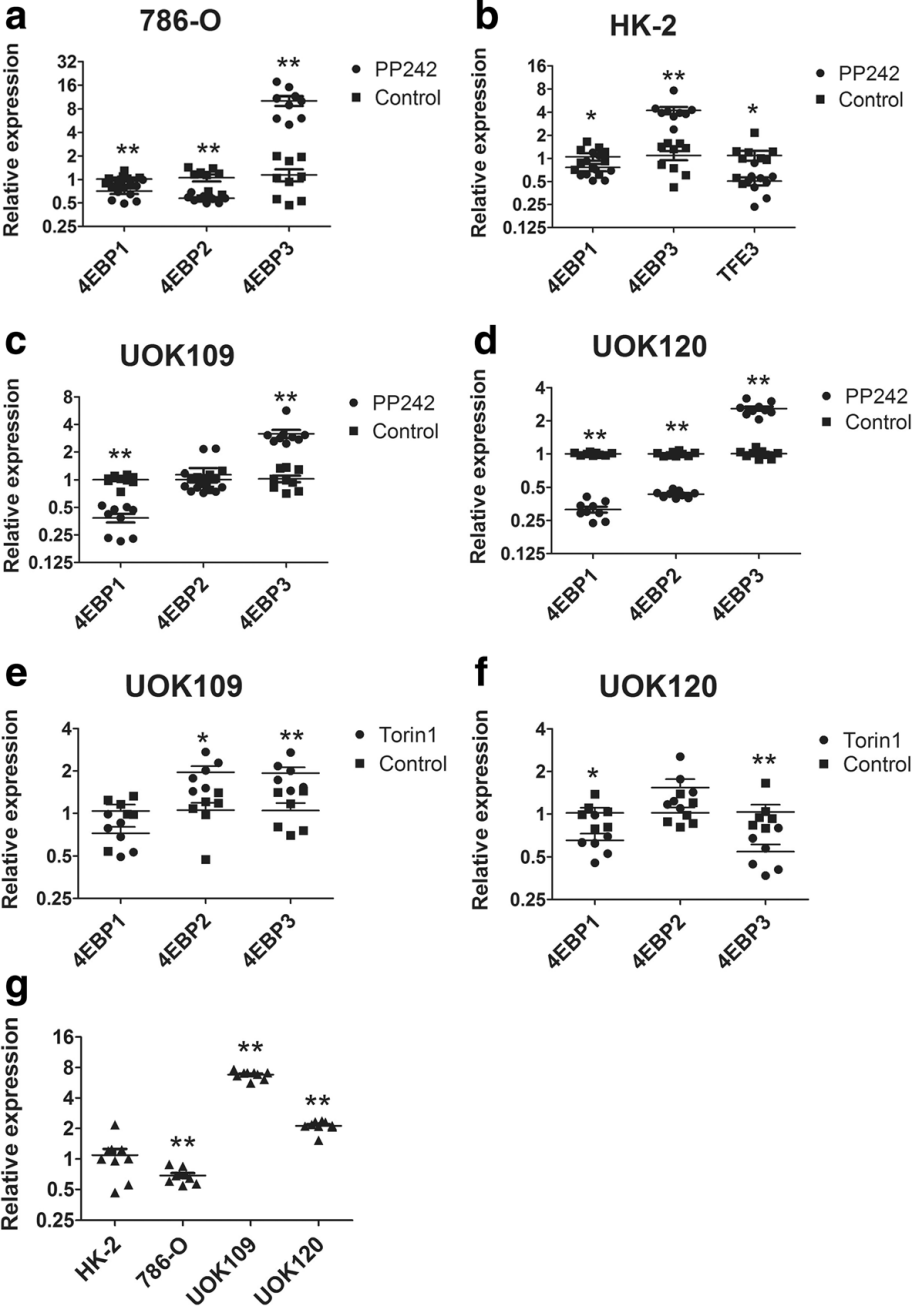
TFE3 fusions promote the expression of 4EBP3 after mTORC1 inhibition. (**a** to **d**) 786-O, HK-2, UOK109 and UOK120 cells were treated with mTOR inhibitor (PP242) or DMSO (Control) for 24 h. The expression of 4EBPs were determined by q-PCR, and GAPDH was used as an internal control. (**e**, **f**) The expression of 4EBPs were detected by q-PCR after 24 h treatment with mTOR inhibitor (Torin1) or left untreated (Control) in UOK109 and UOK120 cells. (**g**) The fold-change of 4EBP3 in 786-O, UOK109 and UOK120 cells compared with HK-2. Data were presented as the mean ± SEM. *P < 0.05, **P < 0.01. The unpaired two-tailed Student’s *t*-test was used to calculate *P* value

It is reported that TFE3 mediates the transcriptional initiation of 4EBP3 in response to mTORC1 inhibition [25]. To study the function of 4EBP3, we examined the effect of mTOR inhibitors (PP242 and Torin1) on the expression of 4EBP3 in 786–0, HK-2, UOK109 and UOK120 cell lines. Prolonged treatment of renal cell lines with mTOR inhibitors resulted in an increase in 4EBP3 at the mRNA level (Fig. 9a-f). Furthermore, we found that fold-change of 4EBP3 in UOK109 and UOK120 were significantly reduced compared with HK-2 and 786-O cells (Fig. 9g). These findings suggested that TFE3 fusions promote the expression of 4EBP3 after mTOR inhibition, while the effect of mTOR inhibitor on TFE3 fusions was diminished in UOK109 and UOK120.

## Discussion

Since the discovery of TFE3 gene fusions, the mechanism underlying the oncogenic effects of these mutations in kidney largely remains unknown. As with other fusion proteins involving transcription factors, promoter substitution appears to be the key molecular event with TFE3 fusions, causing abnormal TFE3 protein activity. All these fusion isoforms become fused with housekeeping genes containing stronger promoters than normal TFE3. In this study, we found that the expression of NonO-TFE3 and PRCC-TFE3 fusions were definitely increased in UOK109 and UOK120 cells compared with the HK-2. In addition, the overexpressed TFE3 fusions showed strong nuclear retention.

According to literature reports, subcellular localization of TFE3 affected its function as a transcription factor. And a recent study showed that stage-specific subcellular relocalization of TFE3 contributes to developmental progression of pluripotent epiblast [26]. TFE3 nuclear translocation is regulated by several signals including mTORC1, ER stress, mitochondrial damage, and pathogen infection [27, 28]. In fully fed cells, TFE3 is recruited to lysosomes through phosphorylation at Ser321 by activating mTORC1 and is retained in the cytosol by the cytosolic chaperone 14–3-3 proteins. Following starvation, lysosomal stress and other conditions inhibit mTORC1, TFE3 is rapidly translocated to the nucleus and activates gene transcription. In contrast to previous studies, we found that TFE3 fusions were capable of escaping from the regulation of mTORC1. Our results revealed that TFE3 fusions had lost its cytosol retention and were mainly accumulated in the nucleus in UOK109 and UOK120 cells. In the cytoplasm, the interaction between TFE3 fusions and 14–3-3 proteins rarely occurred in UOK109 and UOK120 cells. We further demonstrated that TFE3 fusions failed to be localized on the lysosomal surface and were mainly localized in the nucleus. Besides, the TFE3 segments expressed in 293 T cells showed evident cytosolic retention, while the full TFE3 were localized both in the nucleus and the cytoplasm. Bioinformatics analysis revealed that the fused TFE3 gene of UOK109 cells contained 6–10 exons and the fusion TFE3 gene of UOK120 contained 4–10 exons [14]. The TFE3 exon 1 was invariably absent and the universally retained region of the TFE3 gene (6–10 exons) corresponded to a 280 aa C-terminal peptide, which includes the bHLH/LZ dimerization/DNA-binding domain and the putative nuclear localization signal (NLS). Within the fusion *PRCC* sequence in the UOK120 cells, another NLS can be identified (KPKKRK at position 137) at the N-terminal side of the fusion [29]. Consequently, *PRCC-TFE3* contains both NLS sequence which can promote its translocation into the nucleus [30]. The NLS also existed in the N-terminal of the fusion *NonO* sequence in UOK109. The *NonO-TFE3* results in fusion of almost the entire splicing factor protein to the C-terminal part of TFE3 containing the DNA-binding domain [3]. Therefore, the *NonO-TFE3* contained one NLS which also could lead to strong nuclear translocation. In addition, the first 30 N-terminal residues of TFE3, which were absent in the TFE3 fusion proteins, were both necessary and sufficient for interaction with active Rag heterodimers. Although the TFE3 fusions in UOK109 and UOK120 retained the phosphorylation site at Ser321, they lost the first 30 residues making them unavailable for the lysosome recruitment under mTORC1 inhibition. Taken together, the TFE3 fusion proteins could not be regulated by mTORC1 mainly as a results of losing the lysosomal localization signal. The nuclear retention of TFE3 fusions may be attributed to the retained NLS at the C-terminal of the fusion peptide.

The phosphorylation of multiple conserved amino acids mediates TFE3 subcellular localization and protein stabilization. The mTOR kinase was shown to phosphorylate specific serine residues in TFE3 and to play a major role in the regulation of TFE3 subcellular localization [10, 13]. However, in our study the TFE3 fusions were not controlled by the mTOR signaling pathway and were completely accumulated in the nucleus. Therefore, we turned our attention to other phosphorylation sites on TFE3. Previous studies indicated that TFEB can be phosphorylated by GSK3β at residues Ser134 and Ser138, which are highly conversed between TFE3 and TFEB, leading to cytoplasmic retention [31]. In UOK120 cells, phosphorylation of these residues through activation of GSK3β could distinctly increase TFE3 fusions in cytoplasm and decrease their nuclear retention. In addition, the dissociated TFE3 could be dephosphorylated by PKCβ or AKT at C-terminal in the cytoplasm leading to TFE3 ubiquitination and degradation [31–33]. In the HK-2 cells, inhibition of ubiquitination degradation increased TFE3 expression in the cytoplasm. Taken together, phosphorylation of TFE3 fusions by GSK3β promotes its cytoplasmic retention and dissociated TFE3 fusions in the cytoplasm can be degraded through the ubiquitination pathway. All these could provide potential therapeutic targets for decreasing the nuclear retention of TFE3 fusions in Xp11.2 tRCCs.

A recent study revealed that TFE3 can activate gene expression associated with autophagy and lysosomal biogenesis which promote cellular survival during starvation [10]. In this study, we demonstrated that the overexpressed TFE3 fusions could promote the expression of lysosomal biogenesis genes. Furthermore, the ChIP-seq analysis indicated that TFE3 fusions still maintained their regulation of lysosomal biogenesis in the UOK109 cells. Lysosomes are the primary degradation organelles in cells. Lysosomes receive extracellular materials destined for degradation through endocytosis, whereas intracellular components reach lysosomes mainly through autophagy [34]. The increase of lysosomes may lead to frequent biomolecule degradation and recycling. Thus, cells become more sensitive to cellular energy homeostasis. In addition, the ChIP-seq also revealed that TFE3 fusions could regulate cellular responses to hypoxia stress, which could improve cancer cell resistance to hypoxia and thus promote cancer growth. Therefore, these pathogenic mechanisms of TFE3 fusions may be potentially be exploited for the development of therapeutic targets.

## Conclusions

In summary, we observed that overexpressed TFE3 fusions in Xp11.2 tRCC were capable of escaping from the control of the mTOR signaling pathway and showed evident nuclear retention. Inhibition of Gsk3β could increase cytoplasm retention of TFE3 fusions. In addition, TFE3 fusions could bind to target gene promoters as wild-type TFE3, promoting the expression of genes including lysosomal genes and hypoxia stress related genes, which could improve cell resistance against an extreme environment. Unveiling of these pathogenic mechanisms of TFE3 fusions may benefit the development of new cancer.

## Additional files

Additional file 1: **Supplement S1.** The primer sequences of mRNAs applied in this work. (TIF 176 kb)

Additional file 2: **Supplement S2.** (a) The fusion types of three renal carcinoma cell lines were detected by PCR. (b, c) In HK-2 and 786-O cells, expression of TFE3 (red) was examined by immunofluorescence microscopy after 24 h treatment with PP242. Nuclei were stained with DAPI (blue). Scale bar, 50 μm. (TIF 1403 kb)

Additional file 3: **Supplement S3.** Transcript-protein map of TFE3 aligned with *NonO-TFE3* and *PRCC-TFE3* fusions. Multiple sequence alignment highlight the phosphorylation site between wild-type TFE3, *NonO-TFE3* fusions and *PRCC-TFE3* fusions. The breakpoint of the two fusion types were showed in blue words. The phosphorylation sites and the 14–3-3 proteins binding site of TFE3 were showed in red words. (PDF 75 kb)

Additional file 4: **Supplement S4.** The visualization data of ChIP-seq. Peaks were called using MACS version 2, with q-value 6set to 0.05. The horizontal axis of this chart is genomic location and the vertical axis represents bigwig. (TIF 1777 kb)

Additional file 5: **Supplement S5.** Predicted target genes of *NonO-TFE3* in UOK109 cells from ChIP-seq. E-box sequence and distance from transcription start sites were analyzed using UCSC Genome Bioinformatics software. TSS, transcription start site. TTS, transcription terminal site. (XLSX 102 kb)

Additional file 6: **Supplement S6.** Predicted target genes of *PRCC-TFE3* in UOK120 cells from ChIP-seq. E-box sequence and distance from transcription start sites were analyzed using UCSC Genome Bioinformatics software. TSS, transcription start site. TTS, transcription terminal site. (XLSX 29 kb)

## Abbreviations

CCK-8: Cell counting kit-8
ChIP: Chromatin immunoprecipitation
GO: Gene Ontology
Gsk3β: Glycogen synthase kinase 3 beta
KEGG: Kyoto Encyclopedia of Genes and Genomes
LAMP2: Lysosome-associated membrane protein 2
mTOR: mechanistic target of rapamycin
qRT-PCR: Quantitative reverse transcription polymerase chain reaction
TFE3: Transcription factor binding to IGHM enhancer 3
tRCC: translocation renal cell carcinoma
