# Supplementary figures and images for: TFE3 fusions escape from controlling of mTOR signaling pathway and accumulate in the nucleus promoting genes expression in Xp11.2 translocation renal cell carcinomas
Source: J Exp Clin Cancer Res. 2019 Mar 8;38:119. doi: 10.1186/s13046-019-1101-7 (PMC6408813; doi:10.1186/s13046-019-1101-7)

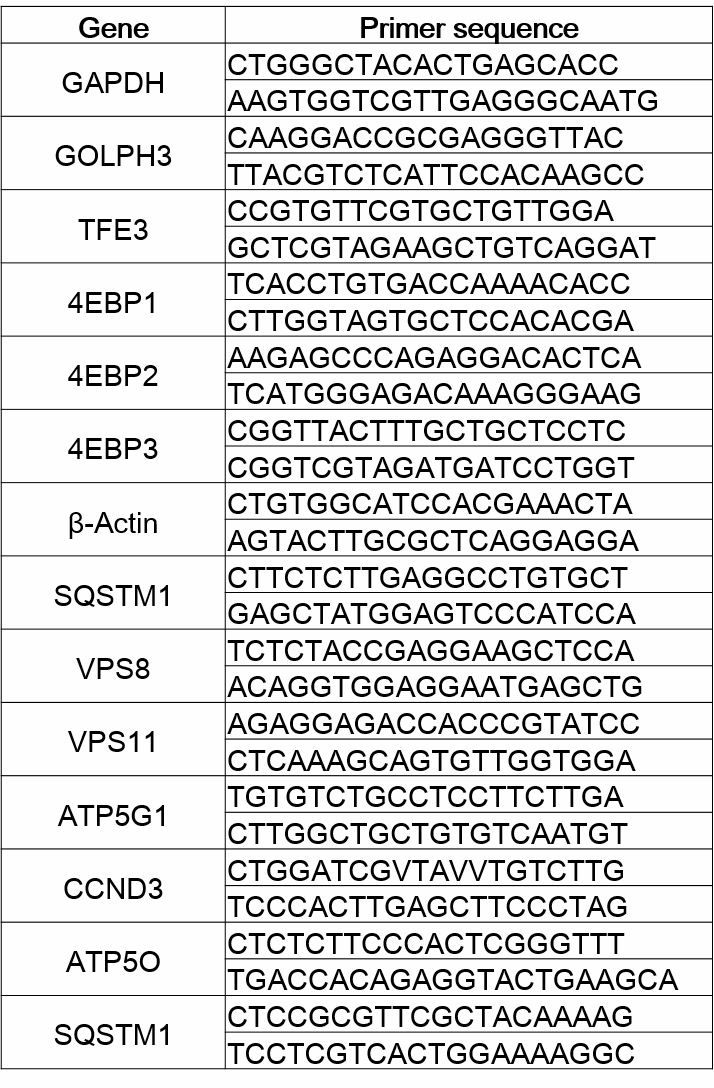

Supplement: Supplementary file 1 — Supplement S1. The primer sequences of mRNAs applied in this work. (TIF 176 kb) [file 13046_2019_1101_MOESM1_ESM.tif]
