# Supplementary material for: TFE3 fusions escape from controlling of mTOR signaling pathway and accumulate in the nucleus promoting genes expression in Xp11.2 translocation renal cell carcinomas
Source: J Exp Clin Cancer Res. 2019 Mar 8;38:119. doi: 10.1186/s13046-019-1101-7 (PMC6408813; doi:10.1186/s13046-019-1101-7)
Supplement: Supplementary file 3 — Supplement S3. Transcript-protein map of TFE3 aligned with NonO-TFE3 and PRCC-TFE3 fusions. Multiple sequence alignment highlight the phosphorylation site between wild-type TFE3, NonO-TFE3 fusions and PRCC-TFE3 fusions. The breakpoint of the two fusion types were showed in blue words. The phosphorylation sites and the 14–3-3 proteins binding site of TFE3 were showed in red words. (PDF 75 kb) [file 13046_2019_1101_MOESM3_ESM.pdf]

```
NonO-TFE3          MQSNKTFNLEKQNHTPRKHHQHHHQQQHHQQQQQQPPPPPIPANGQQASSQNEGLTIDLK 60
Wild-typeTFE3      -MSHA-----------------------AE-----PA--RDGVEASAEGPRA--VFVLLE 27
PRCC-TFE3          -MSLVAYA----------------SSDESEPDEAEPEPEEEEAVAPTSGPALGGLFASLP 43
                     *                          :     *      . .   .     :   *

NonO-TFE3          NFRKPGEKTFTQRSRLFVGNLPPDITEE-EMRKLFEKYGKAGEVFIHKDKGFGFIRLETR 119
Wild-typeTFE3      ERRPADSAQLLSLNSLLP--E-SGIVADIELENVL------------DPDSFYELKSQPL 72
PRCC-TFE3          --APKGP-------ALLP--PPPQMLAPAFPPPLL------------LPPPTGDPRLQ-- 78
                        .         *:       :        ::                    : :

                                                                     Rag binding
NonO-TFE3          TLAEIAKVELDNMPLRGKQLRVRFACHSASLTVRNLPQYVS----NELLEEAFSVFGQVE 175
Wild-typeTFE3      PLRSSLPISLQATPATPATLSA-----SSSAGGSRTPAMSSSSSSRVLLRQ------QLM 121
PRCC-TFE3          -PPPPLPFGLGGFPPPPGVSPA-----E-AAGV--------------------------- 104
                          . *  *      .     . :

NonO-TFE3          RAVVIVDDR-G-RPSGKGIVEFSGKPAAR-KAL---DRCSEGSFLLTTFPRPVT-VEPMD 228
Wild-typeTFE3      RAQAQEQERRERREQAAAA-PF-PSPAPASPAISVVG-VSAGGHTL-SRPPPA------Q 171
PRCC-TFE3          ---------------GEGL-GL-GLPSPRGPGLNLPPPIGGAGPPL-GLPKPKKRKEPVK 146
                                  . .   :   *:    .:      . ..   *   * *       .

                             PRCC-TFE3 breakpoint
NonO-TFE3          QLDDEEGLPEKLVIKNQQFHKEREQPPRFAQPGSFEYEYAMRWKALIEMEKQQQDQVDRN 288
Wild-typeTFE3      --------VPREVL---KVQTHLENPTRY------HLQQARR------------QQVKQY 202
PRCC-TFE3          IA-----APELHKG---DVQTHLENPTRY------HLQQARR------------QQVKQY 180
                                    ..:.. *:* *:      .  : * *            :**.:

                                                                        Gsk3β   mTOR
NonO-TFE3          IKEA-REKLEMEMEAARHEHQVMLMRQDLMRRQEELRRMEELHNQEV------QKRKQLE 341
Wild-typeTFE3      LSTTLGPKLAS---------QALTPPPGP-ASAQPLPAPEAAHTTGPTGSAPNSPMALLT 252
PRCC-TFE3          LSTTLGPKLAS---------QALTPPPGP-ASAQPLPAPEAAHTTGPTGSAPNSPMALLT 230
                   :. :   **           *.:    .     : *   *  *.         .     *

                                                             NonO-TFE3 breakpoint
NonO-TFE3          LRQE--EERRRREEE----MRRQQEEMMRRQQEGFK-GTFPDALPVSGNLLDVYSSQGVA 394
Wild-typeTFE3      IGSSSEKEIDDVIDEIISLESSYNDEMLSYLPGGTTGLQLPSTLPVSGNLLDVYSSQGVA 312
PRCC-TFE3          IGSSSEKEIDDVIDEIISLESSYNDEMLSYLPGGTTGLQLPSTLPVSGNLLDVYSSQGVA 290
                   : ..  :*     :*        ::**:     * .   :*.:*****************

                    mTOR and 14-3-3 binding
NonO-TFE3          TPAITVSNSCPAELPNIKREISETEAKALLKERQKKDNHNLIERRRRFNINDRIKELGTL 454
Wild-typeTFE3      TPAITVSNSCPAELPNIKREISETEAKALLKERQKKDNHNLIERRRRFNINDRIKELGTL 372
PRCC-TFE3          TPAITVSNSCPAELPNIKREISETEAKALLKERQKKDNHNLIERRRRFNINDRIKELGTL 350
                   ************************************************************

NonO-TFE3          IPKSSDPEMRWNKGTILKASVDYIRKLQKEQQRSKDLESRQRSLEQANRSLQLRIQELEL 514
Wild-typeTFE3      IPKSSDPEMRWNKGTILKASVDYIRKLQKEQQRSKDLESRQRSLEQANRSLQLRIQELEL 432
PRCC-TFE3          IPKSSDPEMRWNKGTILKASVDYIRKLQKEQQRSKDLESRQRSLEQANRSLQLRIQELEL 410
                   ************************************************************

NonO-TFE3          QAQIHGLPVPPTPGLLSLATTSASDSLKPEQLDIEEEGRPGAATFHVGGGPAQNAPHQQP 574
Wild-typeTFE3      QAQIHGLPVPPTPGLLSLATTSASDSLKPEQLDIEEEGRPGAATFHVGGGPAQNAPHQQP 492
PRCC-TFE3          QAQIHGLPVPPTPGLLSLATTSASDSLKPEQLDIEEEGRPGAATFHVGGGPAQNAPHQQP 470
                   ************************************************************

NonO-TFE3          PAPPSDALLDLHFPSDHLGDLGDPFHLGLEDILMEEEEGVVGGLSGGALSPLRAASDPLL 634
Wild-typeTFE3      PAPPSDALLDLHFPSDHLGDLGDPFHLGLEDILMEEEEGVVGGLSGGALSPLRAASDPLL 552
PRCC-TFE3          PAPPSDALLDLHFPSDHLGDLGDPFHLGLEDILMEEEEGVVGGLSGGALSPLRAASDPLL 530
                   ************************************************************

                            PKCβ   PKCβ
NonO-TFE3          SSVSPAVSKASSRRSSFSMEEES      657
Wild-typeTFE3      SSVSPAVSKASSRRSSFSMEEES      575
PRCC-TFE3          SSVSPAVSKASSRRSSFSMEEES      553
                   ***********************
```
